# Short-term spark or long-term flame? Sustaining academic psychiatric departments

**DOI:** 10.1192/bjo.2026.10975

**Published:** 2026-02-23

**Authors:** Richard A. Laugharne, Derek K. Tracy, Dinesh Bhugra, Rohit Shankar

**Affiliations:** Cornwall Partnership NHS Foundation Trust, Truro, UK; Cornwall Intellectual Disability Equitable Research, University of Plymouth, Truro, UK; Institute of Psychiatry, King’s College London, UK; South London and Maudsley NHS Foundation Trust, London, UK

**Keywords:** Evidence-based mental health, history of psychiatry, psychological medicine, service development, patients and service users

## Abstract

Over the past two decades, the number of academic psychiatrists in the UK has declined by more than a third, despite an expansion in medical schools and growth in most other medical academic specialties. Drawing on direct experience of establishing a new academic unit, we argue that the long-term sustainability of academic psychiatry departments is critical for service quality, innovation and talent development. This paper outlines the structural, cultural and strategic factors needed to create academic units that endure and flourish beyond individual careers, enabling better integration of research and clinical practice.

**Figure:**
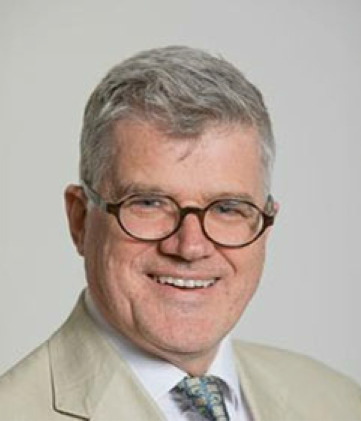

Academic psychiatry in the UK is facing a sustained decline.^1^ The number of full-time equivalent (FTE) academic psychiatrists has fallen from 330 to 206 over the past 20 years, even as medical schools have increased from 30 to 46.^2^ This trend contrasts with the growth in academic posts in most other specialties. Table 1 provides direct comparisons between specialties. It is primarily focused on UK medical schools staffing and does not include National Health Service (NHS) and other non-medical university numbers but these are expected to be very low. While new psychiatric academic units are occasionally established, in our experience many fail to persist beyond the leadership of a single individual.

**Table 1:** The number of clinical academic staff in the UK over time by specialty (FTE)^3^

Specialty	2004	2005	2006	2007	2008	2009	2010	2011	2012	2013	2014	2015	2016	2017	2018	2019	2020	2021	2022	2023
Anaesthetics	60.4	61.4	60.5	59.3	54.7	56.4	49.4	43.2	43.8	44.4	45.1	45.1	43.4	42.8	46.3	49.1	52.5	46.7	55.4	55.4
Emergency medicine	2.0	6.1	0.5	0.0	0.0	4.4	7.5	8.0	10.5	8.9	5.5	5.5	6.0	6.0	10.7	10.5	1.0	1.0	1.0	18.5
General practice	126.8	158.1	156.6	159.4	159.1	167.1	151.8	169.0	163.7	175.9	165.1	177.4	173.6	169.9	177.2	184.5	185.6	170.1	192.0	202.0
Infection/Microbiology	49.5	51.2	56.4	57.0	57.3	68.8	78.6	68.7	68.0	75.4	71.5	61.7	70.4	61.8	65.2	70.3	61.2	61.9	73.4	73.4
Medical education	21.5	18.8	13.7	16.0	21.1	12.2	14.5	18.0	16.1	10.7	10.6	10.2	12.3	27.9	25.1	24.3	28.4	23.3	29.5	32.6
Obstetrics and gynaecology	176.1	110.8	98.2	106.9	98.8	104.2	100.9	85.6	90.4	99.8	95.1	99.7	98.2	87.8	88.5	78.7	79.3	76.9	80.7	99.8
Occupational medicine	12.0	10.0	11.2	14.0	14.0	12.8	11.4	8.2	7.8	6.6	5.6	5.8	4.0	2.0	0.4	0.2	0.4	0.0	0.0	0.0
Oncology	131.1	110.3	97.0	109.8	119.8	117.8	125.5	130.4	127.5	131.9	139.7	140.9	131.1	141.6	142.8	146.2	146.8	144.9	139.0	143.5
Ophthalmology	25.6	29.7	31.7	29.1	29.3	27.5	29.2	35.2	35.4	41.7	44.2	48.0	45.1	46.9	40.1	40.0	41.9	39.6	41.0	41.0
Other	9.7	33.3	55.6	53.0	60.9	49.8	44.8	43.1	52.0	23.4	34.8	25.5	26.3	65.5	130.0	23.5	22.7	21.9	34.7	34.7
Paediatrics and child health	98.8	195.4	179.7	178.5	175.0	181.4	189.3	173.8	171.0	163.6	168.7	152.9	165.3	177.4	175.9	179.5	179.1	165.7	170.9	159.9
Pathology	194.1	172.9	174.4	157.6	147.8	153.7	138.2	135.3	136.1	127.0	126.7	105.3	93.3	88.7	88.4	86.2	83.1	77.9	74.1	71.9
Physician specialties	924.1	954.9	946.5	959.5	1003.9	1029.5	1053.1	1040.2	1020.2	1045.1	1031.1	1017.2	1023.3	1022.6	1014.8	1003.8	958.6	957.2	954.7	954.7
Public health medicine	156.0	137.7	138.9	150.2	141.8	153.4	149.6	156.8	150.9	152.6	148.7	156.0	139.5	144.2	136.3	135.8	123.4	110.3	119.3	119.3
Radiology	35.1	31.7	36.8	37.6	41.7	38.0	40.1	42.6	40.2	40.5	40.6	44.3	41.8	38.8	41.9	37.0	38.5	32.3	37.2	35.5
Surgery	201.7	215.8	224.6	228.5	216.0	210.7	216.6	218.5	212.1	215.8	217.0	200.8	199.5	189.9	203.8	208.5	208.4	216.5	214.2	212.1

FTE, full time equivalent.

This paper poses three interrelated questions: does it matter whether academic psychiatry departments come to existence? Does it matter if they are sustained over 20 to 50 years, rather than 5–10? If so, what strategies can promote their long-term viability?

## The case for existence and sustainability

Critchley et al argue that clinical academics play a central role in generating the evidence base for medical innovation.^2^ Furthermore, clinical services that are research-active are associated with better patient outcomes.^4^ Academic psychiatry fosters a culture of critical inquiry, which is essential for advancing care. Without this, services risk becoming complacent, adhering to outdated norms without challenge or innovation.

Previous UK government policy has favoured concentration of academic capacity in a few high-performing institutions. These large, well-established centres offer economies of scale and critical mass and are vital in sustaining academic psychiatry. However there has been a decline of smaller academic units and therefore an academic career can be restricted to certain geographical areas. For smaller units, the retirement or departure of a single academic leader can precipitate a unit’s collapse.

An evolutionary model, where academic units emerge, flourish and fade, may suit some disciplines. However, in psychiatry, the absence of sustained academic environments limits research exposure for trainees and deprives services of the benefits of innovation. Most importantly, patients in under-researched settings may experience poorer outcomes.

Academic psychiatrists must compete with a growing cadre of non-medical mental health researchers, often psychologists, social scientists and health economists who often have few or no clinical responsibilities and can focus exclusively on research productivity. We contend, however, that clinically active psychiatrists remain uniquely placed to advance the medical care of patients through clinically informed, evidence-based research. Clinically active academic psychiatrists can enrich the research agenda and further its relevance to front-line care. Clinically active researchers have the unique skills to identify sensitive, relevant clinical challenges requiring independent enquiry and can deliver these comprehensively.^5,6^

We therefore argue for a distributed yet non-duplicative network of sustainable academic psychiatry departments across the UK. Such a network could support research-rich cultures in diverse services, enhance care quality and provide the foundation for cross-fertilisation and developing future academic leaders.

## The lifecycle of academic units

Academic units often begin organically, with an individual psychiatrist initiating small projects, establishing collaborations and attracting funding. As output grows, the unit may receive university recognition and attract trainees, eventually becoming a small department. However, these developments typically hinge on a single influential academic’s presence. When that person leaves, projects, grants and momentum may quickly dissipate.

## Strategies for sustaining academic units

### Succession planning

Long-term sustainability requires deliberate succession strategies so that reliance on a single academic leader does not become a point of weakness. This includes identifying and nurturing early- and mid-career academics, building distributed leadership and ensuring that knowledge, networks and resources are not concentrated in a single individual.

### Embedding a research culture

Creating a culture of critical thinking is vital. Resident doctors and early-career psychiatrists need structured opportunities to develop ideas into research, supported by mentors and practical resources. Not all ideas will lead to outputs, but a permissive and encouraging environment is essential. It is important to invest in several potential academic resident psychiatrists so that some might emerge as academic leaders, while others will be academically productive while remaining in the NHS. This range of synergies between the university and the NHS is essential in creating a depth in the relationship between the two institutions.

### Bridging cultural divides

‘Culture eats strategy for breakfast.’^7^ Universities and the NHS operate on divergent cultures. The former is performance-driven and competitive; the latter emphasises risk aversion and service continuity. Clinical academics must navigate both systems, advocating for academic excellence within the NHS and clinical relevance within universities. This is no small challenge and needs both university-based psychiatrists and NHS-based psychiatrists to understand each other and work together to bridge the contrasting cultures. As we witnessed in the research for the COVID pandemic, when this works it can genuinely lead to world-class research. Transparent communication about expectations and success criteria is essential to avoid disillusionment. Clinical academics offer the university a greater chance of clinically relevant research which can have direct impact on clinical care. It is also important that funding awarding committees are not dominated by non-clinical academics so that clinical relevance and impact have greater emphasis in awarding funding.

### Leveraging virtual networks

The post-pandemic shift to remote working has enabled wider collaboration and offers radically new opportunities. Video conferencing has made it feasible to build academic units that transcend geographical boundaries. The UK’s Research Delivery Network (formerly Clinical Research Network) further facilitates multi-site studies and enables distributed research communities. This has created far greater opportunities for individuals and smaller units to collaborate on projects with larger units, which will have the positive benefit of creating a wider dispersal of research opportunities and funding for smaller units.

### International collaboration

The demands of large-scale studies and the new technologies to harness large data-sets for cohort studies also offer new opportunities of international collaborations which are attractive to far-seeing universities. Clinical academics are in a good position to collaborate with colleagues as they have established networks which can be leveraged.

### Developing a triumvirate skillset

Academic psychiatrists should be supported to develop expertise in three domains: clinical care, research scholarship and entrepreneurial income generation. The ability to secure funding from diverse sources is essential for resilience and has not been encouraged as much as it could have been. Performance metrics should reflect this tripartite model, encompassing clinical impact, grant income and scholarly output.

### Defining a core focus

Successful academic units often coalesce around a defined clinical and research niche, considering the ‘place’, uniqueness and potential duplication of this with other national and international centres. A clear identity supports collaboration, funding success and visibility. While diversification is possible, a core area of expertise provides coherence and direction. Creating networks of researchers active in the core focus will seed a research culture more widely.

### Intersectional research

Similar to international collaborations, there is an increasing need in the UK for intersectional psychiatric research, particularly across social, genetic and technological fields. It could be that psychiatric academic units develop based on where specific intersectional expertise for this exists, for example in artificial intelligence and neurodevelopmental research.^8^

### Maintaining patient-centred values

Academic activity must be anchored in patient benefit. All decisions should be guided by the question: how will this improve care? Genuine co-production with service users should be embedded in research processes, moving beyond tokenism to authentic partnership. Relationships with patients and fellow clinicians are great gifts psychiatrists have to bring to research communities.

### Realism about academic careers

Universities will only invest in academics who deliver outputs. This reality must be acknowledged. Productivity will be rewarded, and underperformance may lead to withdrawal of support. Academic clinicians are fortunate in having a clinical role to fall back on, but sustaining an academic post requires ongoing performance. However, adequate funding streams need to be established and maintained. NHS trusts and universities need to support clinicians who spend half their time in clinical work and half in research with long-term tenure. These posts are an asset and not a luxury, and their benefit needs to be demonstrated through the evidence of better patient outcomes, better staff recruitment and greater job satisfaction.

### Recognising ethnicity, diversity and gender talent blindspots

Much of the meaningful psychiatric research emerges from economically developed countries. Academia in the UK in general and in psychiatry in particular has shown to be a male gender dominated field. Due to the competitive nature of the field, such as tight grant submission deadlines and need to ‘publish or perish’ attitudes, this has possibly had an impact disproportionately on female psychiatrists wanting to take time off for maternity and family reasons.^9^ A recent publication has not only explored this gender gap but provides reasonable bottom-up solutions to help bridge it.^10^ Similarly, little thought has been given to also facilitate systemic ethnic inclusion, particularly of first-generation immigrants from developing countries, who form a significant part of the clinical workforce, especially those who have past research experience. Course-correction in these inclusivity aspects could improve academic recruitment and sustainability.

## The role of policy and infrastructure

The National Institute for Health and Care Research (NIHR) has recently funded ten Mental Health Research Groups in regions with historically low research activity. This is a welcome initiative. However, long-term sustainability must be a central consideration. The 37.5% drop in FTEs in academic psychiatry between 2004 and 2024 is not uniform across the country.^1^ The established academic institutions such as in London, Oxford, Cambridge and other major metropolitan areas have had little, if any, change.^3^ The big losses in FTE numbers are more associated with geographically distant areas from London. For example, Yorkshire-Humber and the South-West of the UK had losses of 20 and 15%, respectively. This is a direct result of academic units in these areas shutting or winding down due to being dependent on a single person. The success of centres such as the Institute of Psychiatry, Psychology and Neuroscience reflects not only excellence but also sustained strategic investment. There is no reason why similar success cannot be achieved elsewhere, provided vision, infrastructure and leadership are in place.

The erosion of academic psychiatry is not inevitable. Through succession planning, cultural adaptation, strategic focus and policy support, sustainable academic units can be built and maintained. There might be an opportunity through crisis: right now, contemporary healthcare systems are generally in an era of austerity. While this has, and does, lead to cuts in services, it also emphasises the need for more evidence-based interventions, the understanding of local populations and use of good data, and the development of more effective models of care. Such units are vital for innovation, training and most importantly better patient care. We call for a coordinated national strategy to support and sustain academic psychiatry across the UK.

## Data Availability

Data availability is not applicable to this article as no new data were created or analysed in this study.
